# Development of an effective predictive screening tool for prostate cancer using the ClarityDX machine learning platform

**DOI:** 10.1038/s41746-024-01167-9

**Published:** 2024-06-20

**Authors:** M. Eric Hyndman, Robert J. Paproski, Adam Kinnaird, Adrian Fairey, Leonard Marks, Christian P. Pavlovich, Sean A. Fletcher, Roman Zachoval, Vanda Adamcova, Jiri Stejskal, Armen Aprikian, Christopher J. D. Wallis, Desmond Pink, Catalina Vasquez, Perrin H. Beatty, John D. Lewis

**Affiliations:** 1 Department of Surgical Oncology, University of Calgary, Prostate Cancer Centre, Calgary, T2P 1P9 AB Canada; 2 Nanostics Inc., 4550 10230 Jasper Avenue, Edmonton, T5J 4P6 AB Canada; 3 Division of Urology, Department of Surgery, University of Alberta, Kipnes Urology Centre, Edmonton, T6G 1Z1 AB Canada; 4 Department of Oncology, University of Alberta, Edmonton, T6G 2E1 AB Canada; 5 UCLA Health, Westwood Urology 200 Medical Plaza, Suite 140, Los Angeles, CA 90095 USA; 6 James Buchanan Brady Urological Institute, Johns Hopkins University School of Medicine, Baltimore, 21287 MD USA; 7 Department of Urology, 3rd Faculty of Medicine of Charles University and Thomayer University Hospital, Prague, Czech Republic; 8 Department of Surgery, McGill University, Montreal, H3G 2M1 QC Canada; 9 Division of Urology, Department of Surgery, University of Toronto, Toronto, M5T 1P5 ON Canada; 10 Division of Urology, Department of Surgery, Mount Sinai Hospital, Toronto, M5G 1X5 ON Canada; 11 Department of Surgical Oncology, University Health Network, Toronto, ON Canada

**Keywords:** Predictive markers, Prostate cancer

## Abstract

The current prostate cancer (PCa) screen test, prostate-specific antigen (PSA), has a high sensitivity for PCa but low specificity for high-risk, clinically significant PCa (csPCa), resulting in overdiagnosis and overtreatment of non-csPCa. Early identification of csPCa while avoiding unnecessary biopsies in men with non-csPCa is challenging. We built an optimized machine learning platform (ClarityDX) and showed its utility in generating models predicting csPCa. Integrating the ClarityDX platform with blood-based biomarkers for clinically significant PCa and clinical biomarker data from a 3448-patient cohort, we developed a test to stratify patients’ risk of csPCa; called ClarityDX Prostate. When predicting high risk cancer in the validation cohort, ClarityDX Prostate showed 95% sensitivity, 35% specificity, 54% positive predictive value, and 91% negative predictive value, at a ≥ 25% threshold. Using ClarityDX Prostate at this threshold could avoid up to 35% of unnecessary prostate biopsies. ClarityDX Prostate showed higher accuracy for predicting the risk of csPCa than PSA alone and the tested model-based risk calculators. Using this test as a reflex test in men with elevated PSA levels may help patients and their healthcare providers decide if a prostate biopsy is necessary.

## Introduction

Prostate cancer (PCa) is the most common cancer in men and the fifth most common cause of cancer mortality globally^1^. Screening men for PCa using the prostate-specific antigen (PSA) blood test has shown a decrease in PCa-related mortality^2,3^. However, some urological advisory boards still recommend against PSA screening due to overdiagnosis of low-risk disease which causes significant morbidity from prostate biopsies^4,5^. Recent reports indicate that the decline in PSA screening has led to increases in late-stage diagnosis and PCa mortality^6–8^. However, the advent of new PCa screening tools in the last decade such as risk calculators and biofluid biomarker tests, has led to the greater adoption of risk-based screening approaches for identifying clinically significant prostate cancer (csPCa; grade group (GG) ≥ 2) which requires treatment^7,9^. Risk-based screening methods, now recommended by the European Urology Association (EUA), adds another test if PSA levels are high^10,11^. Low-cost, highly specific csPCa tests are crucial after an elevated PSA to prevent over-diagnosis, over-treatment, and unnecessary health complications and costs^7,12–16^.

Multiparametric MRI (mpMRI) has been recommended for use when prostate cancer is suspected after a previous negative biopsy and as a standard of care diagnostic tool before biopsy to help detect csPCa and reduce overdiagnosis of non-csPCa^11,17,18^. Studies suggest mpMRI with targeted biopsy is better than systematic biopsies for correct diagnoses of csPCa but that its accuracy is also highly dependent on operator expertise^19,20^. Other studies indicate substantial intra-reader and inter-reader variability with mpMRI outputs, and some men with csPCa are missed^21–23^. In the US, the cost of routine mpMRI prior to biopsy is estimated to be $3 billion annually, roughly 15% of the entire cost of managing PCa, thus it would be more cost effective if only patients with prostate cancer would undergo mpMRI for the benefits of targeted biopsies^24–27^. In addition, patients in many countries have limited access to mpMRI^24,25,27^. Early PCa diagnosis guidelines recommend using PSA plus a fluid (blood or urine) biomarker test or risk prediction model to improve the sensitivity and specificity for csPCa before biopsy^20,28,29^. PCa risk models include ERSPC-calculator 3, the PCa Prevention Trial Risk Calculator (PCPTRC), the Prostate Biopsy Collaborative Group (PBCG) plus diagnostic biomarker tests such as 4Kscore, prostate health index (PHI), and the Stockholm3 test^2,30–34^.

Prostate biopsy, the gold standard PCa diagnosis procedure, is invasive, painful, and can cause adverse effects, psychological stress, lower patient quality of life and increase healthcare costs^12,35–38^. Many studies have shown that prostate biopsies are linked with over-detection of clinically insignificant PCa and potentially unnecessary biopsies^39–41^.

Here, we describe the development of ClarityDX, a predictive analysis platform able to generate risk scores from biomarker data and clinical features using our optimized machine learning approach. We also describe the utility of the platform with the development of a test called ClarityDX Prostate to predict csPCa using readily available patient clinical characteristics and approved laboratory tests. This test aims to improve the selection of men requiring mpMRI and prostate biopsies from the men who do not require these procedures, thereby reducing patient adverse events and overall healthcare costs.

## Results

### Clinical features

Notable clinical features that differed between clinical sites (Table 1) included, prior negative biopsies were less than 8% from UA and UC while 50% of TUH participants had a prior negative biopsy. All participants from UCLA and TUH had pre-biopsy MRI results versus 3.4% from UC. Although relatively few UA participants had pre-biopsy MRIs, the detection rate of csPCa was not increased through the use of pre-biopsy MRI (4/15 (27%) csPCa with pre-biopsy MRI vs 169/427 (40%) csPCa without pre-biopsy MRI; *p*-value = 0.42).

**Table 1:** Patient characteristics by clinical site

	Training			Validation	
	University of California, Los Angeles	University of Calgary	Johns Hopkins University	University of Alberta	Thomayer University Hospital
**Patients, n**	1394	442	355	938	319
**Age, yr, median (IQR)**	65 (59–70)	64 (58–68)	65 (60–70)	63 (58–68)	64 (57–68)
**Ethnicity, n (%)**					N/A
**African American**	71 (5.1%)	6 (1.4%)	66 (19%)	38 (4.1%)	
**Asian**	85 (6.1%)	48 (11%)	8 (2.3%)	88 (9.4%)	
**Hispanic**	8 (0.57%)	6 (1.4%)	7 (2.0%)	17 (1.8%)	
**Native American**	1 (0.072%)	1 (0.23%)	0 (0%)	34 (3.6%)	
**White**	935 (67%)	376 (85%)	263 (74%)	745 (79%)	
**Other**	63 (4.5%)	5 (1.1%)	6 (1.7%)	15 (1.6%)	
**Unknown**	231 (17%)	0 (0.0%)	5 (1.4%)	1 (0.11%)	
**Family history PCa, n (% abnormal)**			N/A		N/A
**Yes**	341 (24%)	118 (27%)		290 (31%)	
**No**	1014 (73%)	301 (68%)		585 (62%)	
**Unknown**	39 (2.8%)	23 (5.2%)		63 (6.7%)	
**Previous negative biopsy, n (%)**					
**Yes**	518 (37%)	34 (7.7%)	59 (17%)	65 (6.9%)	161 (50%)
**No**	876 (63%)	408 (92%)	296 (83%)	872 (93%)	158 (50%)
**Unknown**	0 (0%)	0 (0%)	0 (0%)	1 (0.11%)	0 (0%)
**Number of previous negative biopsies, mean (SD)**	0.50 (0.73)	0.090 (0.34)	0.22 (0.60)	0.083 (0.32)	0.96 (1.2)
**DRE, n (% abnormal)**					
**Yes**	187 (13%)	144 (33%)	70 (20%)	225 (24%)	25 (7.8%)
**No**	835 (60%)	249 (56%)	264 (74%)	367 (39%)	294 (92%)
**Unknown**	372 (27%)	49 (11%)	21 (5.9%)	346 (37%)	0 (0%)
**PSA, ng/ml, median (IQR)**	6.6 (4.8–9.4)	7.3 (5.6–9.9)	6.2 (4.7–8.4)	7.4 (5.6–11)	6.2 (4.2–9.4)
**Free PSA, ng/ml, median (IQR)**	0.98 (0.66–1.5)	1.2 (0.85–1.8)	0.90 (0.60–1.4)	1.1 (0.74–1.5)	0.88 (0.57–1.5)
**% Free PSA, median (IQR)**	15 (10–20)	16 (11–21)	14 (10–20)	14 (9.5–19)	15 (11–19)
**Pre-biopsy MRI, n (%)**					
**PI-RADS** ≤ **2**	354 (25%)	3 (0.68%)	50 (14%)	30 (3.2%)	52 (16%)
**PI-RADS** ≥ **3**	1040 (75%)	12 (2.7%)	192 (54%)	166 (18%)	267 (84%)
**Unknown**	0 (0%)	427 (97%)	113 (32%)	742 (79%)	0 (0%)
**Biopsy results, n (%)**					
**Negative**	561 (40%)	142 (32%)	95 (27%)	275 (29%)	158 (50%)
**Grade Group 1**	253 (18%)	127 (29%)	92 (26%)	224 (24%)	45 (14%)
**Grade Group 2**	266 (19%)	125 (28%)	78 (22%)	291 (31%)	50 (16%)
**Grade Group 3**	118 (8.5%)	30 (6.8%)	36 (10%)	107 (11%)	26 (8.2%)
**Grade Group 4**	84 (6.0%)	7 (1.6%)	15 (4.2%)	10 (1.1%)	23 (7.2%)
**Grade Group 5**	112 (8.0%)	11 (2.5%)	39 (11%)	31 (3.3%)	17 (5.3%)

*IQR* Interquartile range, *PCa* Prostate cancer, *SD* Standard deviation, *DRE* Digital rectal exam, *PSA* Prostate Specific Antigen, *N/A* Not available.

Clinical features were individually assessed for predicting csPCa in the training cohort (Table 2). Clinical features that significantly differed between patients without and with csPCa in the training cohort included previous negative biopsy (34% vs 20%, *p*-value < 0.0001), number of previous negative biopsies (mean 0.45 vs 0.26, *p*-value < 0.0001), abnormal DRE (11% vs 29%, *p*-value < 0.0001), age (median 63 years vs 66 years, *p*-value < 0.0001), PSA (median 6.2 ng/mL vs 7.7 ng/mL, *p*-value < 0.0001), and % free PSA (17% vs 12%, *p*-value < 0.0001). PSA and % free PSA were the most predictive single features for csPCa (AUC of 0.63 and 0.69, respectively).

**Table 2:** Predictive value of single features in the training cohort for GG ≥ 2 prostate cancer

	Predicting grade group ≥ 2 prostate cancer			
	GG ≤ 1 PCa	GG ≥ 2 PCa	p-value	ROC AUC (CI)
Patients, n	1270 (58%)	921 (42%)		
African American, n (%)	31 (2.4%)	35 (3.8%)	0.076	0.51 (0.50 - 0.51)
Asian, n (%)	93 (7.3%)	48 (5.2%)	0.052	0.51 (0.50 - 0.52)
Hispanic, n (%)	12 (0.94%)	9 (0.98%)	1.0	0.50 (0.50 - 0.50)
Native American, n (%)	1 (0.079%)	1 (0.11%)	1.0	0.50 (0.50 - 0.50)
White, n (%)	923 (73%)	651 (71%)	0.29	0.51 (0.49 - 0.53)
Family history PCa, n (%)	281 (22%)	178 (19%)	0.29	0.51 (0.49 - 0.53)
Prior negative biopsy, n (%)	428 (34%)	183 (20%)	**<0.0001**	0.57 (0.55 - 0.59)
Number of prior negative biopsies, mean (SD)	0.45 (0.73)	0.26 (0.56)	**<0.0001**	0.57 (0.55 - 0.59)
DRE, n (% abnormal)	136 (11%)	265 (29%)	**<0.0001**	0.58 (0.56 - 0.60)
Age, yr, median (IQR)	63 (58 - 68)	66 (61 - 71)	**<0.0001**	0.60 (0.58 - 0.63)
PSA, ng/ml, median (IQR)	6.2 (4.6 - 8.5)	7.7 (5.5 - 11)	**<0.0001**	0.63 (0.61 - 0.65)
Free PSA, ng/ml, median (IQR)	1.0 (0.70 - 1.5)	0.95 (0.66 - 1.5)	0.19	0.52 (0.49 - 0.54)
% Free PSA, median (IQR)	17 (13 - 22)	12 (9.0 - 17)	**<0.0001**	0.69 (0.67 - 0.71)

*GG* Grade group, *PCa* Prostate cancer, *ROC AUC* Receiver operating characteristic area under the curve, *CI* Confidence interval (95%), *DRE* Digital rectal exam, *IQR* Interquartile range, *PSA* Prostate-specific antigen.

Bold values are statistically significant.

### Predictive model development and performance optimization

**Fig. 1:**
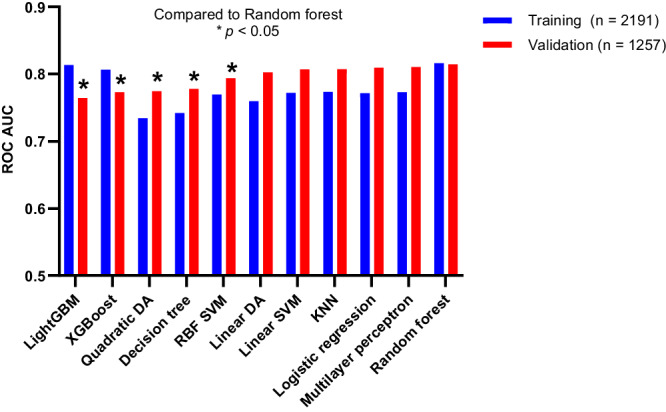
Comparing 11 machine learning algorithms for predicting csPCa defined as grade group ≥2 prostate cancer using ROC AUC values. ROC AUC values were compared using DeLong’s method. GBM: gradient-boosting machine, DA: discriminant analysis, RBF: radial basis function, SVM: support vector machines, KNN: k-nearest neighbors.

Eleven machine learning algorithms were compared for predicting csPCa (Fig. 1). Five features were used for models predicting csPCa including PSA, % free PSA, age, DRE findings, and previous negative biopsy status (yes or no). The models with the highest AUC values (0.81) for the validation cohort included random forest, multilayer perceptron, and logistic regression. Other ensemble decision tree algorithms, including LightGBM and XGBoost models, overfit the training cohort data since they had high AUCs for the training cohort ( >0.8) but the lowest AUCs for the validation cohort ( < 0.78). Only the random forest model was capable of AUCs greater than 0.8 for both the training and validation cohorts. Model parameter optimization was important for clinical performance since not performing this optimization reduced the random forest model AUC value to 0.78 for the validation cohort (data not shown).

Additional feature engineering was performed to model age since it exhibited a non-linear relationship with the probability of csPCa. When plotting patient age and PCa probability in the training cohort, the data better fit an exponential growth equation than a linear relationship (R squared 0.99 vs. 0.95, Supplementary Fig. 1). The age-related csPCa feature used the exponential growth curve to determine the risk of csPCa which was used as an input feature for predictive models. To further improve model performance, 50 different random forest models were created with different training cohort data subsets and calibrated with isotonic regression. The optimized random forest model had higher AUC values than logistic regression with the largest improvement in the training cohort (0.82 vs 0.77, *p*-value < 0.0001, Supplementary Fig. 2).

### Determining feature importance in the optimized model

Feature elimination from the optimized random forest model was used to determine the importance of each feature for predicting csPCa (Supplementary Fig. 3). Model AUC decreased the most when removing % free PSA with AUC values decreasing by 0.055 and 0.045 for the training and validation cohorts (*p*-values < 0.05), respectively. PSA was the next most important feature since removing PSA decreased AUC values by 0.01 and 0.013 in the training and validation cohorts, respectively (*p*-values < 0.05). Individually removing age or the age-related risk of GG ≥ 2 PCa had non-significant AUC value changes for the training and validation cohort. Removing both age features significantly decreased the AUC for both cohorts below 0.792 (*p*-value < 0.05). Both age-based features were kept in the final model since using them better balanced model AUC between the training and validation cohorts. Individually removing DRE and prior negative biopsy features decreased AUC values for both cohorts by at least 0.008, although the decrease was only significant in the training cohort.

### Performance of ClarityDX Prostate model for predicting grade group ≥ 2 prostate cancer in the training and validation cohorts

**Fig. 2:**
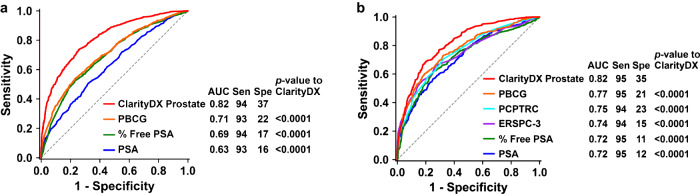
ROC curves for ClarityDX Prostate, prostate cancer risk calculators, % Free PSA, and PSA when predicting csPCa defined as grade group ≥2 prostate cancer. ROC curves in the training cohort (**a**) and validation cohort (**b**). AUC values were compared using DeLong’s method. ClarityDX Prostate had significantly greater AUC values than all other tested risk calculators and PSA for predicting prostate cancer and csPCa in the training and validation cohorts. Sen: sensitivity, Spe: specificity.

The final optimized calibrated random forest ensemble model was called ClarityDX Prostate and the model prediction for csPCa was called the ClarityDX Prostate Risk Score. Compared to PSA, % free PSA, and risk calculators, ClarityDX Prostate provided the highest AUC value for predicting csPCa for the training and validation cohorts (Fig. 2a, b) with the following ranking by AUC on the validation cohort: ClarityDX Prostate (0.82), PBCG (0.77, *p*-value < 0.0001), PCPTRC (0.75, *p*-value < 0.0001), ERSPC-3 (0.74, *p*-value < 0.0001), % free PSA (0.72, *p*-value < 0.0001), and PSA (0.72, *p*-value < 0.0001). To calculate ClarityDX Prostate sensitivity and specificity, a Risk Score threshold of 25% was used since it provided ≥94% sensitivity in the training and validation cohorts (Tables 3, 4). For both the training and validation cohorts, ClarityDX Prostate had a specificity that was ≥14% (absolute value) higher than PBCG (Table 3) and ≥21% (absolute value) higher than PSA and % free PSA while maintaining a sensitivity equal or greater than the other tests. ClarityDX Prostate AUC values for predicting csPCa were not significantly different for cohorts including or excluding participants with prior negative biopsies with AUC, sensitivity, and specificity values ≥ 0.81, ≥94%, and ≥35%, respectively, for both the training and validation cohorts (Supplementary Table 2).

**Table 3:** Comparison of single features, PBCG, and ClarityDX Prostate for predicting grade group ≥2 prostate cancer in the training cohort

	GG ≤ 1 PCa	GG ≥ 2 PCa	p-value	ROC AUC (CI)	Cutoff	Sensitivity, % (CI)	Specificity, % (CI)	PPV, % (CI)	NPV, % (CI)
Patients, n	1270 (58%)	921 (42%)							
Prior negative biopsy, n (%)	428 (34%)	183 (20%)	**<0.0001**	0.57 (0.55–0.59)		80 (77–83)	34 (31–36)	47 (44–49)	70 (66–74)
DRE, n (% abnormal)	136 (11%)	265 (29%)	**<0.0001**	0.58 (0.56–0.60)		32 (29–35)	85 (83–87)	66 (61–71)	57 (55–60)
Age, yr, median (IQR)	63 (58–68)	66 (61 - 71)	**<0.0001**	0.60 (0.58–0.63)	≥55	93 (91–94)	13 (11–15)	43 (41–46)	70 (64–76)
PSA, ng/ml, median (IQR)	6.2 (4.6–8.5)	7.7 (5.5 - 11)	**<0.0001**	0.63 (0.60–0.65)	≥4.1	93 (91–94)	16 (14–18)	45 (42–47)	76 (70–80)
% Free PSA, median (IQR)	17 (13–22)	12 (9 - 17)	**<0.0001**	0.69 (0.67–0.71)	<24.8	94 (92–95)	17 (15–19)	45 (43–47)	78 (73–83)
PBCG, median (IQR)	25 (16–35)	38 (25 - 54)	**<0.0001**	0.71 (0.68–0.73)	≥14.4	93 (91–94)	22 (20–24)	46 (44–48)	80 (76–84)
ClarityDX Prostate, median (IQR)	30 (21–44)	58 (41 - 79)	**<0.0001**	0.82 (0.80–0.83)	≥25	94 (92–95)	37 (34–40)	52 (50–54)	89 (86–91)

*GG* Grade group, *PCa* Prostate cancer, *ROC AUC* Receiver operating characteristic area under the curve, *CI* Confidence interval (95%), *PPV* Positive predictive value, *NPV* Negative predictive value, *DRE* Digital rectal exam, *IQR* Interquartile range, *PSA* Prostate-specific antigen, *PBCG* Prostate Biopsy Collaborative Group risk calculator.

Bold values are statistically significant.

**Table 4:** Comparison of single features, risk calculators, and ClarityDX Prostate for predicting grade group ≥2 prostate cancer in the validation cohort

	GG ≤ 1 PCa	GG ≥ 2 PCa	p-value	ROC AUC (CI)	Cutoff	Sensitivity, % (CI)	Specificity, % (CI)	PPV, % (CI)	NPV, % (CI)
**Patients, n**	702 (56%)	555 (44%)							
**Prior negative biopsy, n (%)**	158 (23%)	68 (12%)	**<0.0001**	0.55 (0.53–0.57)		88 (85–90)	23 (20–26)	47 (44 –50)	70 (64–76)
**DRE, n (% abnormal)**	73 (10%)	177 (32%)	**<0.0001**	0.66 (0.63–0.69)		46 (40–50)	86 (83–89)	71 (65–76)	68 (64–71)
**Age, yr, median (IQR)**	62 (56–66)	65 (61–70)	**<0.0001**	0.65 (0.62–0.68)	≥55	94 (92–96)	16 (14–19)	47 (44–50)	78 (70–84)
**PSA, ng/ml, median (IQR)**	6.2 (4.8–8.4)	9.1 (6.5–15)	**<0.0001**	0.72 (0.69–0.74)	≥4.0	95 (95–97)	12 (12–15)	46 (41–48)	78 (74–82)
**% Free PSA, median (IQR)**	16 (12–21)	11 (8–15)	**<0.0001**	0.72 (0.69–0.75)	<25.1	95 (92–96)	11 (8.8–13)	46 (43–49)	72 (63–80)
**ERSPC-3, median (IQR)**	6.0 (3.0–12)	19 (7.0–41)	**<0.0001**	0.74 (0.71–0.77)	≥1.5	94 (92–96)	15 (12–17)	47 (44–50)	76 (68–82)
**PCPTRC, median (IQR)**	9.0 (6.0–12)	14 (10–22)	**<0.0001**	0.75 (0.72–0.78)	≥5.5	94 (92–96)	23 (20–26)	49 (46–52)	84 (78–88)
**PBCG, median (IQR)**	24 (16–37)	46 (31–64)	**<0.0001**	0.77 (0.74–0.79)	≥14.4	95 (92–96)	21 (18–24)	49 (46–52)	83 (77–88)
**ClarityDX Prostate, median (IQR)**	32 (22–47)	63 (44–82)	**<0.0001**	0.82 (0.79–0.84)	≥25	95 (94–97)	35 (32–39)	54 (51–57)	91 (87–94)

*GG* Grade group, *PCa* Prostate cancer, *ROC AUC* Receiver operating characteristic area under the curve, *CI* Confidence interval (95%), *PPV* Positive predictive value, *NPV* Negative predictive value, *DRE* Digital rectal exam, *IQR* Interquartile range, *PSA* Prostate-specific antigen, *ERSPC-3* European Randomized Study of Screening for Prostate Cancer risk calculator 3, *PCPTRC* Prostate Cancer Prevention Trial Risk Calculator, *PBCG* Prostate Biopsy Collaborative Group risk calculator.

Bold values are statistically significant.

**Fig. 3:**
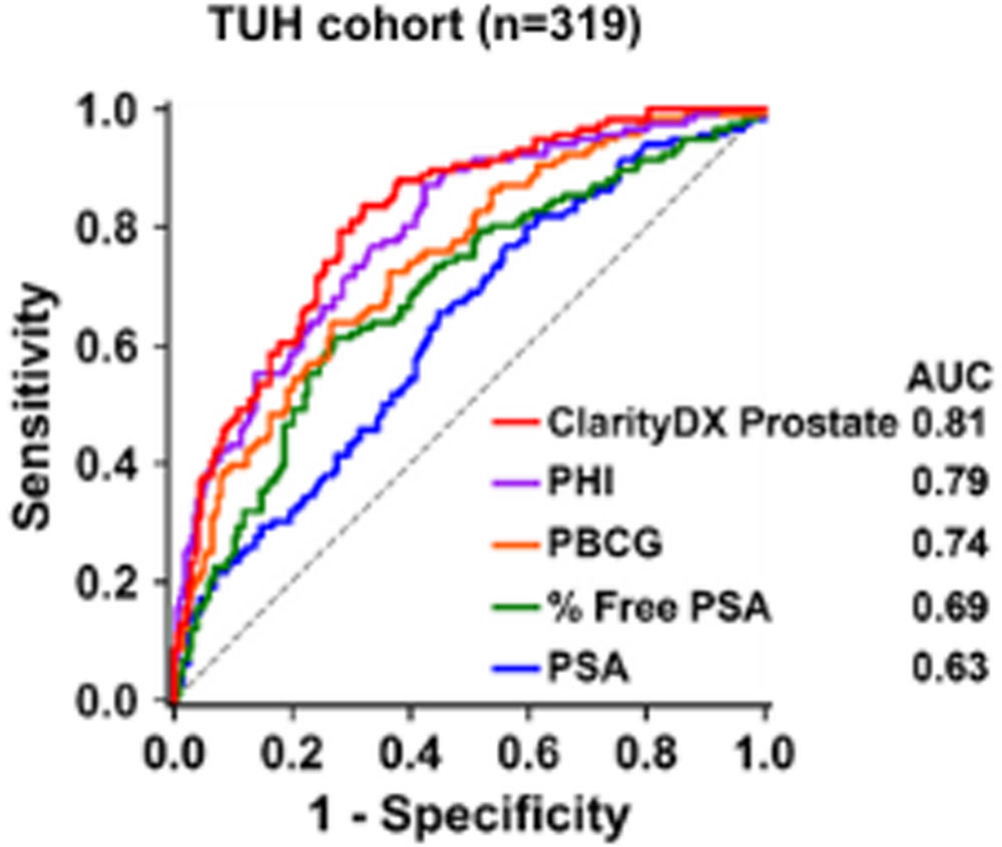
ROC curves for ClarityDX Prostate, PHI, PBCG, % Free PSA, and PSA when predicting grade group ≥2 prostate cancer for Thomayer University Hospital patients.

Patients enrolled at TUH had PHI tests results which were compared to ClarityDX Prostate (Fig. 3). For predicting csPCa, ClarityDX Prostate had a higher AUC value than PHI (0.81 vs 0.79) although they were not significantly different given the relatively small cohort size (n = 319, *p*-value = 0.46).

### ClarityDX Prostate and mpMRI

ClarityDX Prostate was compared to mpMRI for predicting csPCa using patients in the training and validation cohorts who had pre-biopsy mpMRI data. Assuming a positive mpMRI was PI-RADS ≥ 3, mpMRI had sensitivities of 90% and 96% in the training and validation cohorts, respectively (Table 5). Using thresholds for ClarityDX Prostate which matched mpMRI sensitivity, ClarityDX Prostate had at least 7% higher specificity, 3% higher positive predictive value, and 2% higher negative predictive value for both the training and validation cohorts.

**Table 5:** Comparison of MRI and ClarityDX Prostate for predicting grade group ≥2 prostate cancer in the training and validation cohorts

	Training (n = 1651)					Validation (n = 515)			
Test	Sensitivity	Specificity	PPV	NPV		Sensitivity	Specificity	PPV	NPV
MRI PI-RADS ≥ 3	90	35	50	84		96	25	49	90
ClarityDX Prostate^a^	90	46	54	87		96	32	52	92

*PPV* Positive predictive value, *NPV* Negative predictive value, *MRI* Magnetic resonance imaging, *PI-RADS* Prostate imaging reporting & data system

^a^Threshold modified for each cohort to match MRI PI-RADS ≥ 3 sensitivity.

### ClarityDX Prostate Risk Score threshold and prediction of csPCa

When assuming a prostate biopsy was performed if the ClarityDX Prostate Risk Score was ≥25%, an estimated 35% of prostate biopsies could be avoided in the validation cohort while missing 11% GG ≥ 1, 4.5% GG ≥ 2, 3.7% GG ≥ 3, 2.5% GG ≥ 4, and 2.1% GG 5 PCa (Table 6). Similar results were observed in the training cohort with a threshold of ≥25% predicting 94% of csPCa and missing 14% GG ≥ 1, 6.3% GG ≥ 2, 3.5% GG ≥ 3, 3.4% GG ≥ 4, and 2.5% GG 5 PCa (Supplementary Table 3).

**Table 6:** Prostate cancers found, missed, and biopsies avoided using ClarityDX Prostate model in the validation cohort

Thresholds	GG ≥ 1 PCa found	GG ≥ 1 PCa missed	GG ≥ 2 PCa found	GG ≥ 2 PCa missed	GG ≥ 3 PCa found	GG ≥ 3 PCa missed	GG ≥ 4 PCa found	GG ≥ 4 PCa missed	GG >5 PCa found	GG >5 PCa missed	Biopsies avoided^a^	Unnecessary biopsies avoided^b^
	**n (%)**	**n (%)**	**n (%)**	**n (%)**	**n (%)**	**n (%)**	**n (%)**	**n (%)**	**n (%)**	**n (%)**	**n (%)**	**n (%)**
5	823 (99.9%)	1 (0.1%)	555 (100%)	0 (0.0%)	214 (100%)	0 (0.0%)	81 (100%)	0 (0.0%)	48 (100%)	0 (0.0%)	7 (0.6%)	7 (1.0%)
10	813 (98.7%)	11 (1.3%)	555 (100%)	0 (0.0%)	214 (100%)	0 (0.0%)	81 (100%)	0 (0.0%)	48 (100%)	0 (0.0%)	38 (3.0%)	38 (5.4%)
15	796 (96.6%)	28 (3.4%)	551 (99.3%)	4 (0.7%)	213 (99.5%)	1 (0.5%)	81 (100%)	0 (0.0%)	48 (100%)	0 (0.0%)	98 (7.8%)	94 (13.4%)
20	779 (94.5%)	45 (5.5%)	545 (98.2%)	10 (1.8%)	210 (98.1%)	4 (1.9%)	80 (98.8%)	1 (1.2%)	47 (97.9%)	1 (2.1%)	160 (12.7%)	150 (21.4%)
25	732 (88.8%)	92 (11.2%)	530 (95.5%)	25 (4.5%)	206 (96.3%)	8 (3.7%)	79 (97.5%)	2 (2.5%)	47 (97.9%)	1 (2.1%)	272 (21.6%)	247 (35.2%)
30	689 (83.6%)	135 (16.4%)	517 (93.2%)	38 (6.8%)	203 (94.9%)	11 (5.1%)	79 (97.5%)	2 (2.5%)	47 (97.9%)	1 (2.1%)	361 (28.7%)	323 (46.0%)
35	630 (76.5%)	194 (23.5%)	480 (86.5%)	75 (13.5%)	188 (87.9%)	26 (12.1%)	74 (91.4%)	7 (8.6%)	44 (91.7%)	4 (8.3%)	469 (37.3%)	394 (56.1%)
40	579 (70.3%)	245 (29.7%)	448 (80.7%)	107 (19.3%)	179 (83.6%)	35 (16.4%)	70 (86.4%)	11 (13.6%)	40 (83.3%)	8 (16.7%)	560 (44.6%)	453 (64.5%)
45	519 (63.0%)	305 (37.0%)	409 (73.7%)	146 (26.3%)	165 (77.1%)	49 (22.9%)	62 (76.5%)	19 (23.5%)	37 (77.1%)	11 (22.9%)	655 (52.1%)	509 (72.5%)
50	459 (55.7%)	365 (44.3%)	378 (68.1%)	177 (31.9%)	154 (72.0%)	60 (28.0%)	60 (74.1%)	21 (25.9%)	36 (75.0%)	12 (25.0%)	741 (58.9%)	564 (80.3%)

*GG* Grade group, *PCa* Prostate cancer.

^a^Patients receiving a biopsy but had a ClarityDX Prostate Risk Score below the threshold

^b^Patients with a negative biopsy or grade group 1 PCa with a ClarityDX Prostate Risk Score below the threshold.

### Calibration

**Fig. 4:**
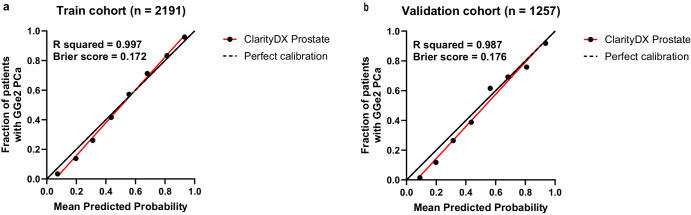
Calibration curves for ClarityDX Prostate model in the training and validation cohorts when predicting grade group ≥2 prostate cancer. Training cohort (**a**) and validation cohort (**b**). Data points fitted with linear regression with R squared values and Brier scores are shown. Calibration curves trend close to the perfect calibration line for training and validation cohorts.

The ClarityDX Prostate model demonstrated good consistency between the predicted and observed probabilities for predicting csPCa with Brier scores of 0.172 and 0.176 for the training and validation cohorts, respectively (Fig. 4a, b). Linear regression equations fit near the perfectly calibrated line with R squared values of 0.997 and 0.987 for the training and validation cohorts, respectively.

### Decision curve analysis

**Fig. 5:**
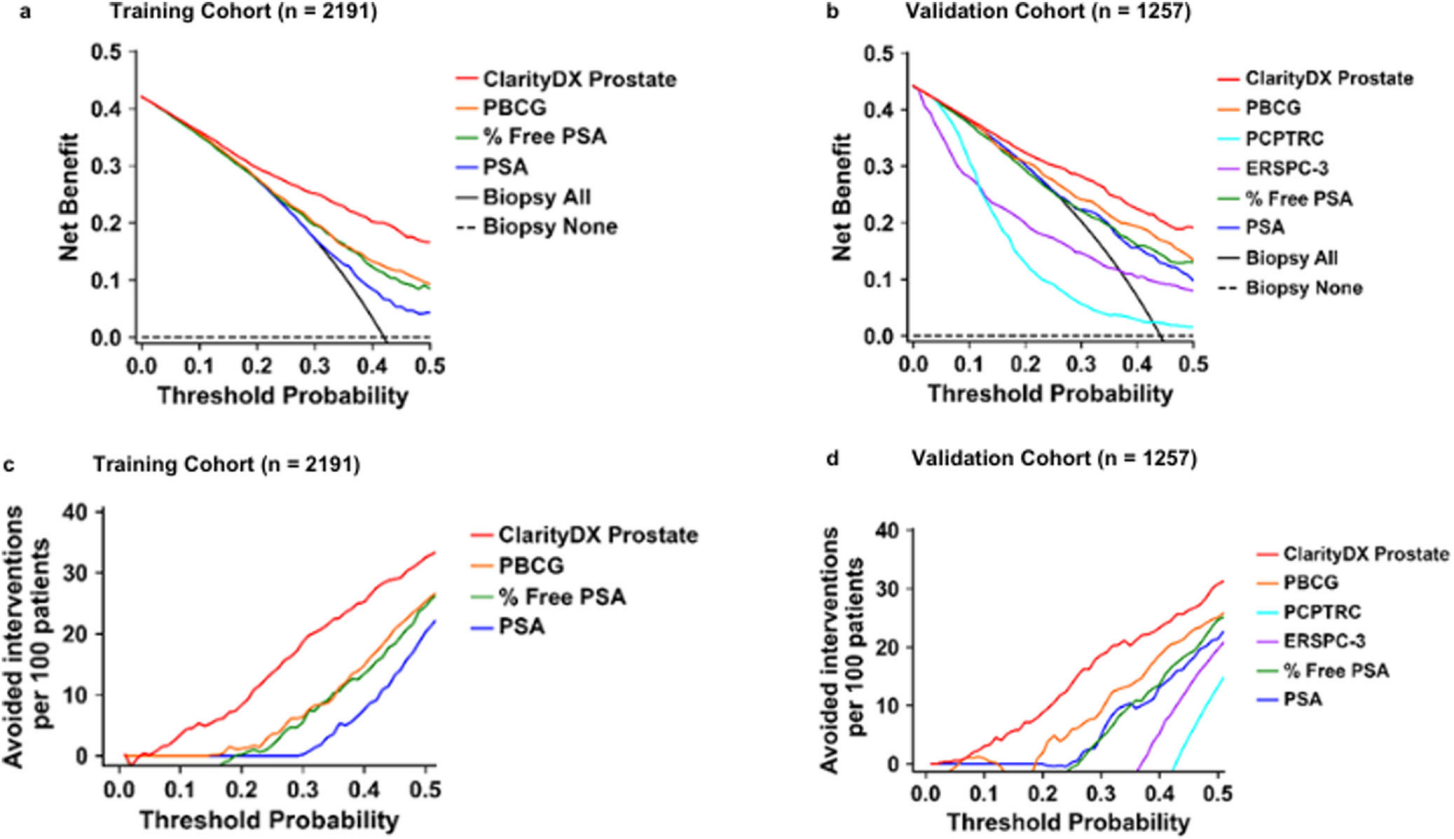
Decision curve analysis of ClarityDX Prostate, prostate cancer risk calculators, % free PSA, and PSA for predicting grade group ≥2 prostate cancer in the training and validation cohorts. Training (**a**, **c**) and validation (**b**, **d**) cohorts. Net benefit (**a**, **b**) and net avoided interventions per 100 patients (**c**, **d**) were analyzed assuming interventions required for grade group ≥2 prostate cancer. For almost all probability thresholds, ClarityDX Prostate outperforms the other tests and models.

Decision curve analysis was performed on ClarityDX Prostate models, risk calculators, % free PSA, and PSA. For both the training and validation cohorts, ClarityDX Prostate demonstrated the highest net benefit and reduction in interventions when predicting csPCa for nearly all probability thresholds between 0.01 to 0.50 (Fig. 5). The recommended ≥25% ClarityDX Prostate threshold is well within the area of highest net benefit for identifying high-risk patients for csPCa.

## Discussion

Using clinical and laboratory data, ClarityDX Prostate predicted the risk of csPCa prior to biopsy in the pre-mpMRI setting. In vitro diagnostic tests with similar outputs to ClarityDX Prostate include PHI, 4Kscore, and Stockholm3^30,31,34^. PHI helps distinguish PCa from benign prostatic conditions and is intended for a more refined population (men aged 50+ with 4.0 to 10.0 ng/mL total PSA, and non-suspicious DRE) than ClarityDX Prostate. The 4Kscore helps detect csPCa in men recommended for biopsy by a urologist; similar to the ClarityDX Prostate intended-use population. Stockholm3 was developed as a general screening test for csPCa although its intended patient population is 45 to 74 years of age and PSA ≥ 1.5 ng/mL^42^. ClarityDX Prostate is a relatively simpler test since it analyzes only two kallikreins compared to PHI, 4Kscore, and Stockholm3 which analyzes three kallikreins, four kallikreins, and five protein markers (including 3 kallikreins) with analysis of ≥100 single nucleotide polymorphisms, respectively.

Cross study comparisons suggest that ClarityDX Prostate may provide similar performance to 4Kscore for predicting csPCa (0.82 vs. 0.80)^43^. Differences in cohorts may affect model performance thus future studies comparing both tests in the same cohort are required. Although 4Kscore has two additional markers not found in ClarityDX Prostate (human kallikrein 2 and intact PSA), they appear to add minimal value based on cross study AUC values. The updated version of the Stockholm3 test has more protein markers than 4Kscore and includes many genetic markers. Based on current results from the SEPTA study, Stockholm3 and ClarityDX Prostate provide similar AUCs for csPCa at 0.82^44^. The minimal number of laboratory tests in ClarityDX Prostate offer several benefits compared to more complicated tests including easier adoption, faster turnaround time, scalability, and cost-effectiveness.

When creating new predictive models with the training cohort in this study, the more advanced machine learning algorithms, such as LightGBM and XGBoost, significantly overfit on the training cohort data and underperformed the linear classifiers when evaluated on a different clinical site. This illustrates the danger of using powerful non-linear models on datasets from different clinical sites. Nested cross-validation of the training and validation data combined allows XGBoost to outperform the linear models (data not shown) but it is unclear how well this model would perform at a different clinical site.

ClarityDX Prostate was based on the random forest model which uses multiple decision trees to predict the probability of csPCa^45^. Each decision tree is a series of questions (e.g., PSA < 3.0 ng/mL) that split based on the answer to additional questions or a ‘leaf’ that contains the predicted class (e.g., csPCa or non-csPCa). A patient’s clinical data is used to determine the path navigated through the tree and the final class predicted for that tree. Since the random forest model has multiple trees, the probability of csPCa is calculated by the proportion of trees that end in a leaf with the csPCa class. An advantage of the random forest model is its ability to identify non-linear relationships between features (e.g., a change in risk only when a feature is below or above a specific threshold value). This change in risk based on feature value is not possible with conventional logistic regression models which requires a constant linear change in log odds for each feature. We have created an ensemble of random forest models which further improves the model stability and performance since it mitigates overfitting due to additional model averaging.

Strengths of this study include derivation of the ClarityDX Prostate model with a relatively contemporary cohort of men from 2009 to 2023. Clinical data was acquired from five clinical sites from three countries, representing a broad and generalizable cohort of patients. Furthermore, free PSA, a relatively uncommon feature in other risk calculators, is used in ClarityDX Prostate which significantly improves model performance. PCPTRC may use free PSA as a feature but it was added to the risk calculator post-hoc using a smaller dataset of 537 men^46^. Additionally, a comprehensive list of machine learning algorithms were optimized for hyperparameters and input features. Finally, the models were validated using different clinical sites from the training data.

Study limitations include the lack of mpMRI data for some patients. Although this lack of mpMRI data does represent real world data in multiple countries where some patients have mpMRI data while others do not. However, pre-biopsy mpMRI data was available for 75% and 41% of patients in the training and validation cohorts, respectively. Additionally, ethnic diversity was relatively low with 67% to 85% of participants self-identifying as White in all clinical sites that provided ethnicity/race data.

Models predicting csPCa typically demonstrate improved diagnostic performance when incorporating prostate volume and PI-RADS score^47–49^. Given the rapid clinical adoption of MRI in the pre-biopsy setting, tests predicting csPCa would likely benefit from the incorporation of these MRI features in the future.

In this study, we show the utility of our optimized machine learning platform ClarityDX to generate models predicting clinically significant disease from patient samples and clinical features. By using prostate cancer-specific blood-based and clinical biomarker data from a 3448-patient cohort and integrating it with the ClarityDX platform, we developed a test to stratify the risk of clinically significant prostate cancer, called ClarityDX Prostate. When predicting high risk cancer in the validation cohort, ClarityDX Prostate showed 95% sensitivity, 35% specificity, 54% positive predictive value, and 91% negative predictive value at a ≥ 25% threshold. Using the test at this threshold could avoid up to 35% of unnecessary prostate biopsies. ClarityDX Prostate was found to be significantly more accurate than PSA and the tested model-based risk calculators.

ClarityDX Prostate is a non-invasive, low risk test that, when used with current standard of care practices, may help physicians improve stratification of at-risk patients requiring more expensive diagnostic procedures such as mpMRI or biopsy.

## Methods

### Study design

Data from multiple academic institutions and hospitals were used to train and validate ClarityDX Prostate including the Alberta Prostate Cancer Research Initiative (APCaRI)^41^ which enrolled Canadian patients from the University of Alberta (UA) and the University of Calgary (UC). Patient data was also acquired from the University of California, Los Angeles (UCLA), USA, Johns Hopkins University (JHU), USA, and Thomayer University Hospital (TUH), Czechia. Patients included in the study 1) were undergoing a prostate biopsy for either elevated PSA or digital rectal exam (DRE) abnormality, 2) did not have a prior PCa diagnosis, and 3) had available data for age, PSA, free PSA, and prostate biopsy results that were being predicted. When comparing ClarityDX Prostate to the PHI test, only TUH patients with PHI and ClarityDX Prostate results were analyzed. The UA and UC sites only enrolled patients between the ages of 40 and 75 years who had total PSA of at least 3 ng/mL within six months of enrollment and excluded those who had a prior cancer diagnosis, except for non-melanoma skin cancer. UCLA, JHU, and TUH sites had no specific enrollment or exclusion criteria related to age, PSA, or prior non-PCa diagnoses. All five sites conducted prostate biopsies between September 2009 and April 2023.

Risk models to predict GG ≥ 2 PCa were derived from the training cohort using data from UCLA, UC, and JHU comprising 2191 eligible patients from a potential 2234 patient cohort. The risk models were fixed and validated on a separate validation cohort from UA and TUH comprising 1257 patients, based on eligibility criteria, from a potential 1562 patient cohort.

### Ethics

UC and UA studies were approved by the Health Research Ethics Board HREBA.CC-18-0241 and 19-0109 respectively. UCLA data was approved by the UCLA Institutional Review Board (IRB #11-001580 and IRB #19-001136). JHU and TUH data was collected as part of approved research projects CR00040216 and HREBA.CC-18-0241 respectively. Informed consent was obtained from all participants and study methodologies conformed to the Declaration of Helsinki standards^50^. ClarityDX Prostate test results were not provided to the clinical sites, and the laboratory personnel performing the tests were blinded for patient characteristics.

### PSA and free PSA tests

For patients recruited from UC and UA, serum samples were collected, and processed as described previously^41^, and shipped to Alberta Precision Laboratories (formally DynaLIFE Medical Labs; Edmonton, Canada) for batch analysis of total PSA and free PSA using a Roche Cobas e801 system. Total PSA and free PSA results were acquired within the 12-week stability range for free PSA and were not subjected to multiple freeze-thaw cycles. For the other clinical sites, PSA and free PSA results were acquired as part of standard clinical practice.

### Prostate biopsies

Prostate biopsies were performed per each hospital’s standard procedure, which predominantly included transrectal ultrasound-guided 12-core biopsies. TUH and UCLA had 12 systematic cores with 3 to 5 additional targeted cores per MRI-identified lesion. Biopsies were analyzed by pathologists at their respective academic hospital centers.

### Covariates and outcome

Models were trained to predict csPCa on a future prostate biopsy using the following base features: age (years), prior negative biopsy status (no or yes encoded as 0 or 1), DRE (normal or abnormal encoded as 0 or 1), total PSA (ng/mL), and free PSA ratio (free PSA / total PSA). These base features were transformed before model training as described below.

### Predictive model input features

Predictive model input features were optimized before model training. Feature scaling, performed by calculating z-scores, was performed for input features for logistic regression, linear and quadratic discriminant analysis, K-nearest neighbors, linear and radial basis function support vector machines, and multiplayer perceptron models. Median feature value imputation (determined from training cohort) was used for all input features except for LightGBM and XGBoost models, which can intrinsically handle missing data. Some model features were engineered such as log PSA and age-related risk of csPCa. The age-related risk feature was determined for each patient based on their age and interpolating risk by plotting the probability of csPCa over age for all training cohort patients where patient ages were grouped into 5 quintiles. Within each quintile, the median age and fraction of patients with csPCa were used for plotting. Data in plots were fit using linear regression or an exponential growth curve using GraphPad Prism 9.5.1 software. Exponential growth equations were used to determine each patient’s age-related risk of csPCa by inputting patient age and solving for risk of csPCa used in model training.

### Machine learning algorithms

Development of the ClarityDX platform compared eleven different machine learning algorithms including logistic regression, linear and quadratic discriminant analysis, k-nearest neighbors, linear and radial basis function support vector machines, single decision tree, random forest, LightGBM, XGBoost, and multilayer perceptron. Models were trained to predict csPCa from biopsy results. The algorithm with the highest receiver operator characteristic area under the curve (ROC AUC) value for the validation cohort was further optimized by performing isotonic regression calibration on models using 5-fold cross-validation. The final ClarityDX Prostate model was composed of an ensemble of 50 calibrated models using the same optimal machine learning algorithm, but base models were created on different random subsets of training data using 5-fold cross-validation.

### Model training process

All predictive models were trained using Python 3.8 with scikit-learn (1.1.2) except the LightGBM and XGBoost models which used the lightgbm (3.3.5) and xgboost (1.7.5) packages, respectively. All models in the ClarityDX platform had hyperparameters optimized for the training cohort by grid searching through all combinations of selected hyperparameters (Table S1). Model hyperparameters that provided the highest ROC AUC value in the training cohort when using 5 repeats of 5-fold cross-validation were used during model training on the entire training cohort for creating the final model. Models created from the training cohort were fixed and used for inference on the validation cohort for cross-clinical site model evaluation. Predictive models were compared to the PBCG risk calculator, which were obtained using an R script, as well as the PCPTRC and ERSPC-3 risk calculators using their web applications^32,51,52^. PCPTRC and ERSPC-3 risk calculator predictions were not available for the training cohort since the UCLA data is in a dedicated environment within UCLA Health with no internet access and risk calculator websites were unavailable.

### Statistical analyses

Unless stated otherwise, all statistics and model calibration curve values were calculated using Python 3.8 software with scikit-learn (1.1.2), scipy (1.9.2), and scikits.bootstrap (1.1.0) packages. When comparing 2 groups, Mann-Whitney U-tests were used for continuous data and Fisher’s exact tests were used for binary categorical data. Statistical significance was determined by *p*-values ≤ 0.05. Clinical features analyzed included total PSA, free PSA, percent free PSA (% free PSA; 100 * free PSA/total PSA), age (years), race/ethnicity (African American, Asian, Hispanic, Native American, White, Other), DRE (normal or abnormal), family history of PCa, previous negative biopsy (yes or no), and the number of previous negative biopsies. Where DRE results were available from a general practitioner physician and a urologist, the urologist DRE results were used. ROC curves were compared by DeLong’s method^53^. A ClarityDX Prostate threshold value of 25% was chosen for identifying high risk of csPCa since it provided a sensitivity close to our goal of 95% and is easy for physicians to remember. The 95% sensitivity goal was chosen to ensure no more than 5% of clinically significant prostate cancers would be missed. Threshold values for other clinical features and risk calculator predictions were set to match the sensitivity of ClarityDX Prostate when possible or a threshold providing a lower sensitivity when not possible to match sensitivities. Confidence intervals were determined with 10,000 resamples of bias-corrected and accelerated bootstrapping. Decision curve analysis was performed using Python 3.9 and the dcurves (1.0.6.2) library.

### Reporting summary

Further information on research design is available in the Nature Research Reporting Summary linked to this article.

### Supplementary information

Supplementary Material

Reporting Summary

## Data Availability

The relevant data generated in this study are available within the article and its supplementary material.

## Code availability

The custom computer code used to generate results reported in the paper is the proprietary confidential information of Nanostics Inc., it cannot be made publicly available.
