# Supplementary material for: Development of an effective predictive screening tool for prostate cancer using the ClarityDX machine learning platform
Source: NPJ Digit Med. 2024 Jun 20;7:163. doi: 10.1038/s41746-024-01167-9 (PMC11190196; doi:10.1038/s41746-024-01167-9)
Supplement: Supplementary file 1 — Supplementary Material [file 41746_2024_1167_MOESM1_ESM.pdf]

Development of an effective predictive screening tool for prostate cancer using the ClarityDX machine learning platform

M. Eric Hyndman[1,2,†], Robert J. Paproski[2,†], Adam Kinnaird[3,4], Adrian Fairey[2,3], Leonard Marks[5], Christian P. Pavlovich[6], Sean A. Fletcher[6], Roman Zachoval[7], Vanda Adamcova[7], Jiri Stejskal[7], Armen Aprikian[2,8], Christopher J.D. Wallis[9,10,11], Desmond Pink[2], Catalina Vasquez[2], Perrin H. Beatty[2], and John D. Lewis[2,4*]

[1]Department of Surgical Oncology, University of Calgary, Prostate Cancer Centre, Calgary, Alberta, Canada, T2P 1P9

[2]Nanostics Inc., 4550 10230 Jasper Avenue, Edmonton, Alberta, Canada, T5J 4P6

[3]Division of Urology, Department of Surgery, University of Alberta, Kipnes Urology Centre, Edmonton, AB, Canada, T6G 1Z1

[4]Department of Oncology, University of Alberta, Edmonton, Alberta, Canada, T6G 2E1

[5]UCLA Health, Westwood Urology 200 Medical Plaza, Suite 140, Los Angeles, CA 90095

[6]James Buchanan Brady Urological Institute, Johns Hopkins University School of Medicine, Baltimore, MD, USA, 21287

[7]Department of Urology, 3rd Faculty of Medicine of Charles University and Thomayer University Hospital, Prague, Czech Republic

[8]Department of Surgery, McGill University, Montreal, Quebec, Canada, H3G 2M1

[9]Division of Urology, Department of Surgery, University of Toronto, Toronto, ON, Canada, M5T 1P5

[10]Division of Urology, Department of Surgery, Mount Sinai Hospital, Toronto, ON, Canada, M5G 1X5

[11]Department of Surgical Oncology, University Health Network, Toronto, ON, Canada

†These authors contributed equally to this work.

Supplementary Material

Supplementary Table 1. Model hyperparameters tested during model optimization.

Supplementary Table 2. ClarityDX Prostate clinical performance including and excluding patients with a prior negative biopsy.

Supplementary Table 3. Prostate cancers found, missed, and biopsies avoided using ClarityDX Prostate model in training cohort.

Supplementary Figure 1. Correlation between age and grade group ≥2 prostate cancer probability in the training cohort. An exponential growth equation has a higher R squared value than linear regression when fitting age and probability of grade group ≥2 prostate cancer.

Supplementary Figure 2. Optimized neural network model has improved receiver operating characteristic area under the curve (ROC AUC) values over default logistic regression models for predicting grade group ≥2 prostate cancer in training and validation cohorts. Receiver characteristic curve area under the curve (ROC AUC) values were compared using DeLong's method.

Supplementary Figure 3. Feature selection by feature elimination for models predicting grade group ≥2 prostate cancer. The free PSA ratio was the most important feature for all models since its

removal caused the largest decrease in model receiver operating characteristic area under the curve (ROC AUC). ROC AUC values were compared to the inclusion of all features using DeLong's method. GG≥2 PCa, grade group ≥2 prostate cancer, DRE: digital rectal exam findings (normal or abnormal), PNB: prior negative biopsies status (yes or no), PSA: prostate specific antigen.

Supplementary Table 1. Model hyperparameters tested during model optimization.

	Hyperparameter values
**Logistic regression**	
**penalty**	l1, l2
**C**	0.1, 0.25, 0.5, 1, 2.5, 5, 10, 25, 50, 100, 250, 500, 1000
**Linear discriminant analysis**	
**solver**	svd, lsqr, eigen
**tol**	0.00001, 0.0001, 0.001
**Quadratic discriminant analysis**	
**tol**	0.00001, 0.0001, 0.001
**K-Nearest Neighbors**	
**n_neighbors**	1, 3, 5, 10, 15, 20, 30, 40, 50, 60, 70, 80, 90, 100
**Linear support vector machines**	
**C**	0.1, 0.25, 0.5, 1, 2.5, 5, 10, 25, 50, 100, 250, 500, 1000
**tol**	0.0001, 0.001, 0.03
**Radial basis function support vector machines**	
**C**	0.1, 0.25, 0.5, 1, 2.5, 5, 10, 25, 50, 100, 250, 500, 1000
**degree**	2, 3
**Decision tree**	
**splitter**	best, random
**min_samples_split**	1, 2, 3, 4, 5, 10, 15, 20, 25
**min_samples_leaf**	1, 2, 3, 4, 5, 10, 15, 20, 25
**Random Forest**	
**criterion**	gini, entropy
**max_depth**	3, 4, 5, 6, 7
**n_estimators**	20, 50, 100, 200, 400, 600
**LightGMB**	
**learning_rate**	0.01, 0.03, 0.1
**num_leaves**	3, 5, 10, 30, 100
**n_estimators**	50, 100, 200
**XGBoost**	
**n_estimators**	50, 100, 200, 400
**max_depth**	1, 2, 3, 4
**learning_rate**	0.01, 0.03, 0.1
**Multilayer Perceptron**	
**hidden_layer_sizes**	1, 2, 3, 4, 5
**activation**	identity, logistic, tanh, relu
**learning_rate_init**	0.3, 0.01, 0.003, 0.001, 0.0003, 0.0001
**alpha**	0.00001, 0.0001, 0.001
**solver**	sgd, lbfgs, adam

Supplementary Table 2. ClarityDX Prostate clinical performance, including and excluding patients with a prior negative biopsy.

Cohort	Patient number	ROC AUC	Sensitivity	Specificity	*p*-value
Training including PNB	2191	0.82	94	37	0.78
Training excluding PNB	1580	0.81	94	36	
Validation including PNB	1257	0.82	95	35	0.91
Validation excluding PNB	1030	0.81	95	39	

ROC AUC: Receiver operating characteristic area under the curve,
PNB: prior negative biopsy

Supplementary Table 3. Prostate cancers found, missed, and biopsies avoided using ClarityDX Prostate model in Training cohort.

Thresholds	GG ≥1 PCa found n (%)	GG ≥1 PCa missed n (%)	GG ≥2 PCa found n (%)	GG ≥2 PCa missed n (%)	GG ≥3 PCa found n (%)	GG ≥3 PCa missed n (%)	GG ≥4 PCa found n (%)	GG ≥4 PCa missed n (%)	GG ≥5 PCa found n (%)	GG ≥5 PCa missed n (%)	Biopsies avoided* n (%)	Unnecessary biopsies avoided** n (%)
5	1381 (99.1%)	12 (0.9%)	919 (99.8%)	2 (0.2%)	451 (99.8%)	1 (0.2%)	267 (99.6%)	1 (0.4%)	162 (100%)	0 (0%)	43 (2.0%)	41 (3.2%)
10	1368 (98.2%)	25 (1.8%)	918 (99.7%)	3 (0.3%)	451 (99.8%)	1 (0.2%)	267 (99.6%)	1 (0.4%)	162 (100%)	0 (0%)	104 (4.7%)	101 (8.0%)
15	1339 (96.1%)	54 (3.9%)	911 (98.9%)	10 (1.1%)	450 (99.6%)	2 (0.4%)	266 (99.3%)	2 (0.7%)	162 (100%)	0 (0%)	183 (8.4%)	173 (13.6%)
20	1278 (91.7%)	115 (8.3%)	891 (96.7%)	30 (3.3%)	447 (98.9%)	5 (1.1%)	264 (98.5%)	4 (1.5%)	161 (99.4%)	1 (0.6%)	332 (15.2%)	302 (23.8%)
25	1195 (85.8%)	198 (14.2%)	863 (93.7%)	58 (6.3%)	436 (96.5%)	16 (3.5%)	259 (96.6%)	9 (3.4%)	158 (97.5%)	4 (2.5%)	529 (24.1%)	471 (37.1%)
30	1095 (78.6%)	298 (21.4%)	821 (89.1%)	100 (10.9%)	424 (93.8%)	28 (6.2%)	252 (94.0%)	16 (6.0%)	154 (95.1%)	8 (4.9%)	742 (33.9%)	642 (50.6%)
35	1000 (71.8%)	393 (28.2%)	769 (83.5%)	152 (16.5%)	406 (89.8%)	46 (10.2%)	243 (90.7%)	25 (9.3%)	153 (94.4%)	9 (5.6%)	925 (42.2%)	773 (60.9%)
40	886 (63.6%)	507 (36.4%)	701 (76.1%)	220 (23.9%)	376 (83.2%)	76 (16.8%)	225 (84.0%)	43 (16.0%)	145 (89.5%)	17 (10.5%)	1101 (50.3%)	881 (69.4%)
45	787 (56.5%)	606 (43.5%)	641 (69.6%)	280 (30.4%)	341 (75.4%)	111 (24.6%)	208 (77.6%)	60 (22.4%)	138 (85.2%)	24 (14.8%)	1257 (57.4%)	977 (76.9%)
50	672 (48.2%)	721 (51.8%)	568 (61.7%)	353 (38.3%)	312 (69.0%)	140 (31.0%)	188 (70.1%)	80 (29.9%)	128 (79.0%)	34 (21.0%)	1418 (64.7%)	1065 (83.9%)

GG: Grade group
PCa: Prostate cancer
* Patients receiving a biopsy but had a ClarityDX Prostate Risk Score below the threshold
** Patients with a negative biopsy or grade group 1 PCa with a ClarityDX Prostate Risk Score below the threshold

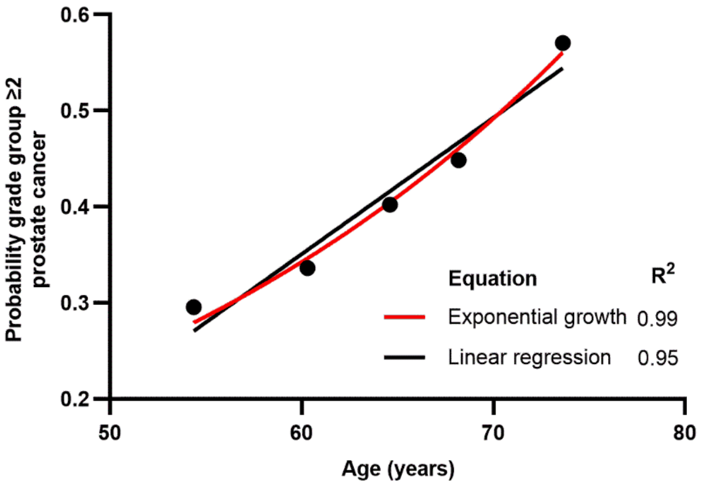

Supplementary Figure 1. Correlation between age and grade group ≥2 prostate cancer probability in the training cohort. An exponential growth equation has a higher R squared value than linear regression when fitting age and probability of grade group ≥2 prostate cancer.

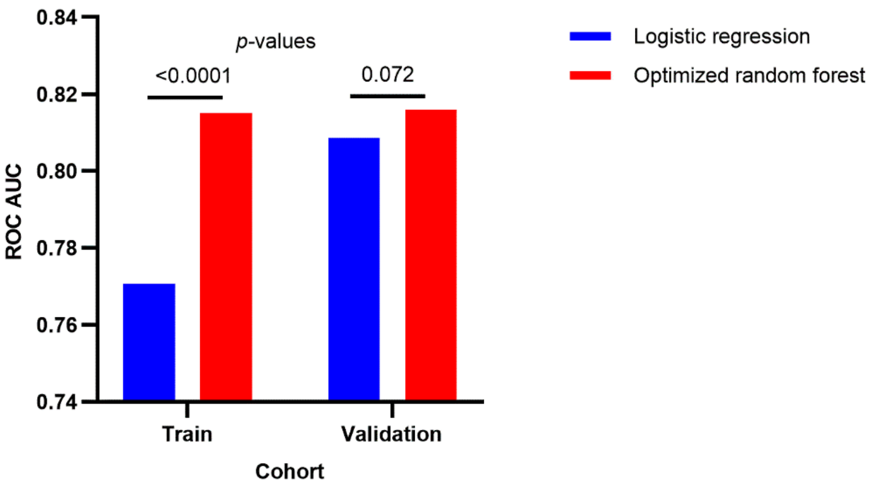

Supplementary Figure 2. Optimized neural network model has improved receiver operating characteristic area under the curve (ROC AUC) values over default logistic regression models for predicting grade group ≥2 prostate cancer in training and validation cohorts. Receiver characteristic curve area under the curve (ROC AUC) values were compared using DeLong's method.

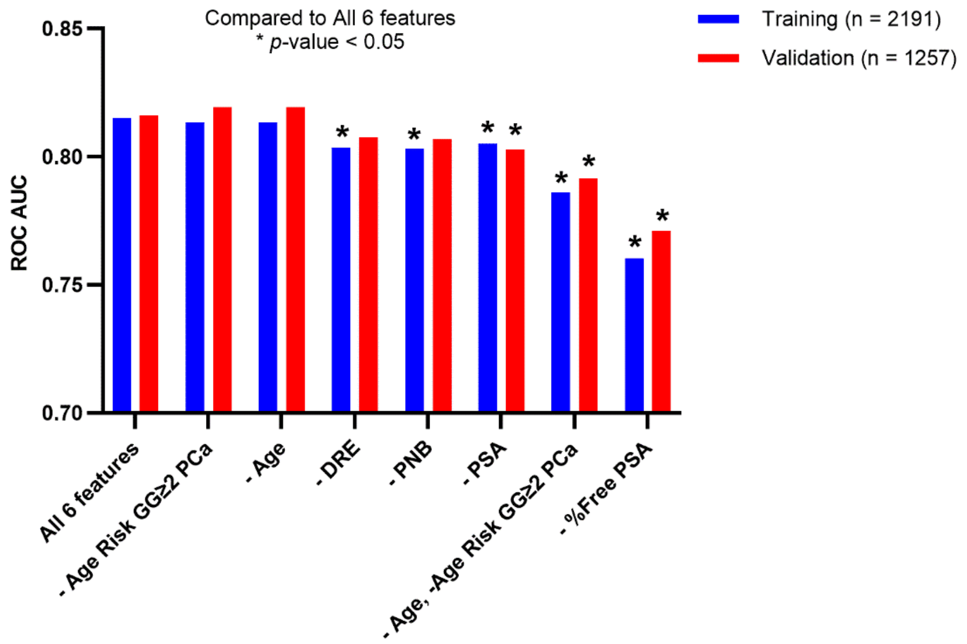

Supplementary Figure 3. Feature selection by feature elimination for models predicting grade group ≥2 prostate cancer. The free PSA ratio was the most important feature for all models since its removal caused the largest decrease in model receiver operating characteristic area under the curve (ROC AUC). ROC AUC values were compared to the inclusion of all features using DeLong's method. GG≥2 PCa, grade group ≥2 prostate cancer, DRE: digital rectal exam findings (normal or abnormal), PNB: prior negative biopsies status (yes or no), PSA: prostate specific antigen.
