# Supplementary material for: Development of an effective predictive screening tool for prostate cancer using the ClarityDX machine learning platform
Source: NPJ Digit Med. 2024 Jun 20;7:163. doi: 10.1038/s41746-024-01167-9 (PMC11190196; doi:10.1038/s41746-024-01167-9)
Supplement: Supplementary file 2 — Reporting Summary [file 41746_2024_1167_MOESM2_ESM.pdf]

Corresponding author(s): John D. Lewis

Last updated by author(s): May 24, 2024

Reporting Summary

Nature Portfolio wishes to improve the reproducibility of the work that we publish. This form provides structure for consistency and transparency in reporting. For further information on Nature Portfolio policies, see our Editorial Policies and the Editorial Policy Checklist.

Statistics

For all statistical analyses, confirm that the following items are present in the figure legend, table legend, main text, or Methods section.

n/a	Confirmed	
☐	☒	The exact sample size (n) for each experimental group/condition, given as a discrete number and unit of measurement
☒	☐	A statement on whether measurements were taken from distinct samples or whether the same sample was measured repeatedly
☐	☒	The statistical test(s) used AND whether they are one- or two-sided<br>*Only common tests should be described solely by name; describe more complex techniques in the Methods section.*
☐	☒	A description of all covariates tested
☐	☒	A description of any assumptions or corrections, such as tests of normality and adjustment for multiple comparisons
☐	☒	A full description of the statistical parameters including central tendency (e.g. means) or other basic estimates (e.g. regression coefficient) AND variation (e.g. standard deviation) or associated estimates of uncertainty (e.g. confidence intervals)
☒	☐	For null hypothesis testing, the test statistic (e.g. F, t, r) with confidence intervals, effect sizes, degrees of freedom and P value noted<br>*Give P values as exact values whenever suitable.*
☒	☐	For Bayesian analysis, information on the choice of priors and Markov chain Monte Carlo settings
☒	☐	For hierarchical and complex designs, identification of the appropriate level for tests and full reporting of outcomes
☒	☐	Estimates of effect sizes (e.g. Cohen's d, Pearson's r), indicating how they were calculated

Our web collection on statistics for biologists contains articles on many of the points above.

Software and code

Policy information about availability of computer code

Data collection	The custom computer code used to generate results reported in the paper is the proprietary confidential information of Nanostics Inc., it cannot be made publicly available.
Data analysis	Development of the ClarityDX platform compared eleven different machine learning algorithms including logistic regression, linear and quadratic discriminant analysis, k-nearest neighbors, linear and radial basis function support vector machines, single decision tree, random forest, LightGBM, XGBoost, and multilayer perceptron. The algorithm with the highest receiver operator characteristic area under the curve (ROC AUC) value for the validation cohort was further optimized by performing isotonic regression calibration on models using 5-fold cross-validation. The final ClarityDX Prostate model was composed of an ensemble of 50 calibrated models using the same optimal machine learning algorithm, but base models were created on different random subsets of training data using 5-fold cross-validation.

For manuscripts utilizing custom algorithms or software that are central to the research but not yet described in published literature, software must be made available to editors and reviewers. We strongly encourage code deposition in a community repository (e.g. GitHub). See the Nature Portfolio guidelines for submitting code & software for further information.

Data

Policy information about availability of data

All manuscripts must include a data availability statement. This statement should provide the following information, where applicable:

- Accession codes, unique identifiers, or web links for publicly available datasets
- A description of any restrictions on data availability
- For clinical datasets or third party data, please ensure that the statement adheres to our policy

The relevant data generated in this study are available within the article and its supplementary material.

Research involving human participants, their data, or biological material

Policy information about studies with human participants or human data. See also policy information about sex, gender (identity/presentation), and sexual orientation and race, ethnicity and racism.

Reporting on sex and gender	Patients included in the study 1) were undergoing a prostate biopsy for either elevated PSA or digital rectal exam (DRE) abnormality, 2) did not have a prior prostate cancer diagnosis, and 3) had available data for age, PSA, free PSA, and prostate biopsy results that were being predicted.
Reporting on race, ethnicity, or other socially relevant groupings	Clinical features analyzed included total PSA, free PSA, percent free PSA (% free PSA; 100 * free PSA/total PSA), age (years), race/ethnicity (African American, Asian, Hispanic, Native American, White, Other), DRE (normal or abnormal), family history of PCa, previous negative biopsy (yes or no), and the number of previous negative biopsies.
Population characteristics	See above.
Recruitment	Patients included in the study 1) were undergoing a prostate biopsy for either elevated PSA or digital rectal exam (DRE) abnormality, 2) did not have a prior prostate cancer diagnosis, and 3) had available data for age, PSA, free PSA, and prostate biopsy results that were being predicted. Data from multiple academic institutions and hospitals were used to train and validate ClarityDX Prostate including the Alberta Prostate Cancer Research Initiative (APCaRI) which enrolled Canadian patients from the University of Alberta (UA) and University of Calgary (UC). Patient data was also acquired from the University of California, Los Angeles (UCLA), USA, Johns Hopkins University (JHU), USA, and Thomayer University Hospital (TUH), Czechia. Patients included in the study 1) were undergoing a prostate biopsy for either elevated PSA or digital rectal exam (DRE) abnormality, 2) did not have a prior PCa diagnosis, and 3) had available data for age, PSA, free PSA, and prostate biopsy results that were being predicted. When comparing ClarityDX Prostate to the PHI test, only TUH patients with PHI and ClarityDX Prostate results were analyzed. The UA and UC sites only enrolled patients between the ages of 40 and 75 years who had total PSA of at least 3 ng/mL within six months of enrollment and excluded those who had a prior cancer diagnosis, except for non-melanoma skin cancer. UCLA, JHU, and TUH sites had no specific enrollment or exclusion criteria related to age, PSA, or prior non-PCa diagnoses. All five sites conducted prostate biopsies between September 2009 and April 2023. Risk models to predict GG ≥2 PCa were derived from the training cohort using data from UCLA, UC, and JHU comprising 2191 eligible patients from a potential 2234 patient cohort. The risk models were fixed and validated on a separate validation cohort from UA and TUH comprising 1257 patients, based on eligibility criteria, from a potential 1562 patient cohort.
Ethics oversight	UC and UA studies were approved by the Health Research Ethics Board HREBA.CC-18-0241 and 19-0109 respectively. UCLA data was approved by the UCLA Institutional Review Board (IRB #11-001580 and IRB #19-001136). JHU and TUH data was collected as part of approved research projects CR00040216 and HREBA.CC-18-0241 respectively. Study methodologies conformed to the Declaration of Helsinki standards 43. ClarityDX Prostate test results were not provided to the clinical sites, and the laboratory personnel performing the tests were blinded for patient characteristics.

Note that full information on the approval of the study protocol must also be provided in the manuscript.

Field-specific reporting

Please select the one below that is the best fit for your research. If you are not sure, read the appropriate sections before making your selection.

☒ Life sciences ☐ Behavioural & social sciences ☐ Ecological, evolutionary & environmental sciences

For a reference copy of the document with all sections, see nature.com/documents/nr-reporting-summary-flat.pdf

Life sciences study design

All studies must disclose on these points even when the disclosure is negative.

Sample size	Risk models to predict GG ≥2 PCa were derived from the training cohort using data from UCLA, UC, and JHU comprising 2191 eligible patients from a potential 2234 patient cohort. The risk models were fixed and validated on a separate validation cohort from UA and TUH comprising 1257 patients, based on eligibility criteria, from a potential 1562 patient cohort.
Data exclusions	The UA and UC sites only enrolled patients between the ages of 40 and 75 years who had total PSA of at least 3 ng/mL within six months of enrollment and excluded those who had a prior cancer diagnosis, except for non-melanoma skin cancer. UCLA, JHU, and TUH sites had no

specific enrollment or exclusion criteria related to age, PSA, or prior non-PCa diagnoses.

Replication	All predictive models were trained using Python 3.8 with scikit-learn (1.1.2) except the LightGBM and XGBoost models which used the lightgbm (3.3.5) and xgboost (1.7.5) packages, respectively. All models in the ClarityDX platform had hyperparameters optimized for the training cohort by grid searching through all combinations of selected hyperparameters (Table S1). Model hyperparameters that provided the highest ROC AUC value in the training cohort when using 5 repeats of 5-fold cross-validation were used during model training on the entire training cohort for creating the final model. Models created from the training cohort were fixed and used for inference on the validation cohort for cross-clinical site model evaluation. Predictive models were compared to the PBCG risk calculator, which were obtained using an R script, as well as the PCPTRC and ERSPC-3 risk calculators using their web applications32,44,45. PCPTRC and ERSPC-3 risk calculator predictions were not available for the training cohort since the UCLA data is in a dedicated environment within UCLA Health with no internet access and risk calculator websites were unavailable.
Randomization	The final ClarityDX Prostate model was composed of an ensemble of 50 calibrated models using the same optimal machine learning algorithm, but base models were created on different random subsets of training data using 5-fold cross-validation.
Blinding	ClarityDX Prostate test results were not provided to the clinical sites, and the laboratory personnel performing the tests were blinded for patient characteristics.

Reporting for specific materials, systems and methods

We require information from authors about some types of materials, experimental systems and methods used in many studies. Here, indicate whether each material, system or method listed is relevant to your study. If you are not sure if a list item applies to your research, read the appropriate section before selecting a response.

Materials & experimental systems

n/a	Involved in the study
☒	☐ Antibodies
☒	☐ Eukaryotic cell lines
☒	☐ Palaeontology and archaeology
☒	☐ Animals and other organisms
☒	☐ Clinical data
☒	☐ Dual use research of concern
☒	☐ Plants

Methods

n/a	Involved in the study
☒	☐ ChIP-seq
☒	☐ Flow cytometry
☒	☐ MRI-based neuroimaging

Plants

Seed stocks	NA
Novel plant genotypes	NA
Authentication	NA
